# Significance of the anatomical relationship between the flexor digitorum longus and sustentaculum tali for reconsideration of the talocalcaneonavicular joint stability mechanism

**DOI:** 10.1038/s41598-022-19543-4

**Published:** 2022-09-08

**Authors:** Masahiro Tsutsumi, Shintarou Kudo, Akimoto Nimura, Keiichi Akita

**Affiliations:** 1 Inclusive Medical Sciences Research Institute, Morinomiya University of Medical Sciences, 1-26-16 Nankokita, Suminoe-ku, Osaka City, Osaka 559-8611 Japan; 2 Department of Clinical Anatomy, Graduate School of Medical and Dental Sciences, Tokyo Medical and Dental University, Tokyo, Japan; 3 Department of Functional Joint Anatomy, Graduate School of Medical and Dental Sciences, Tokyo Medical and Dental University, Tokyo, Japan

**Keywords:** Musculoskeletal system, Orthopaedics

## Abstract

The talocalcaneonavicular joint (TCN-j) is supported by the spring ligament, which has recently been revealed to be part of the joint capsule complex, along with the tendinous sheath of the tibialis posterior and flexor digitorum longus (FDL). Nonetheless, the FDL’s role in TCN-j stability has received limited attention. This study aimed to elucidate the positional relationships between the FDL and sustentaculum tali, which comprises the TCN-j. We hypothesized that the FDL runs medial to the sustentaculum tali, and its course significantly changes from the sitting to the standing position. Six ankles from six body donors were investigated, and seven ankles from seven volunteers were assessed using ultrasonography. The FDL was three-dimensionally located inferomedial to the sustentaculum tali. The FDL tendinous sheath was attached to the sustentaculum tali or connected by the tibialis posterior via the tendinous sheath. Based on the in vivo ultrasound image, the FDL location relative to the sustentaculum tali was maintained; however, the curvature of the FDL course was significantly more prominent in standing than in sitting. The FDL force against the bending moment may prevent the excessive eversion of the foot and aid the conventional spring ligament’s contribution to TCN-j stability for maintaining the longitudinal arch.

## Introduction

Adult acquired flatfoot deformity, recently named as progressive collapsing foot deformity^1,2^, has been conventionally classified based on the tibialis posterior (TP) tendon condition^3^. More recently, assessment of talocalcaneonavicular joint (TCN-j) stability by the spring ligament is considered to be the most important in any adult acquired flatfoot deformity evaluation^2,4–7^. The spring ligament is generally considered as the band connecting the sustentaculum tali and navicular below the head of talus^8^. However, a recent anatomical study revealed that the conventional spring ligament was not a band but is a part of the ankle joint capsule, which forms a complex with the tendinous sheath of the TP and flexor digitorum longus (FDL)^9^. Therefore, the possible role of the FDL in TCN-j stability via the joint capsule complex should also be anatomically reconsidered in order to achieve better adult acquired flatfoot deformity evaluation. However, it has received limited attention because the importance of the TP or so-called spring ligament has always been emphasized more.

According to the anatomy textbooks, the FDL tendon passes behind the bony groove of the medial malleolus with the TP, then turns obliquely forwards and laterally and runs toward the sole of the foot^10^. However, with regard to this running course, the positional relationships between the FDL and sustentaculum tali are not clearly described in past articles and textbooks^11–15^ despite the existence of anatomical textbooks stating that the FDL runs medial to the sustentaculum tali^10,16–18^. This lack of clarity in the positional relationship may also be due to the limited consideration of the role of FDL in TCN-j stability. As the sustentaculum tali is the attachment site of the spring ligament, which is vital in TCN-j stability^2,4–7^, and also receives concentrated stress during weight-bearing^19,20^, the coordination of the sustentaculum tali movement may be vital in TCN-j stability during weight-bearing. However, it also remains unclear whether the weight-bearing stress, such as a posture change from sitting to standing, affects the positional relationship between the FDL and sustentaculum tali.

This study aimed to elucidate the positional relationships between the FDL and sustentaculum tali based on the three-dimensional (3D) image and histological analysis of the anatomic specimen and to visualize its change in the weight-bearing stress by in vivo ultrasound imaging. We hypothesized that the FDL ran medial to the sustentaculum tali, and its course significantly changed from the sitting position to the standing position.

## Results

### 3D image analysis of relationships between FDL and sustentaculum tali

**Figure 1:**
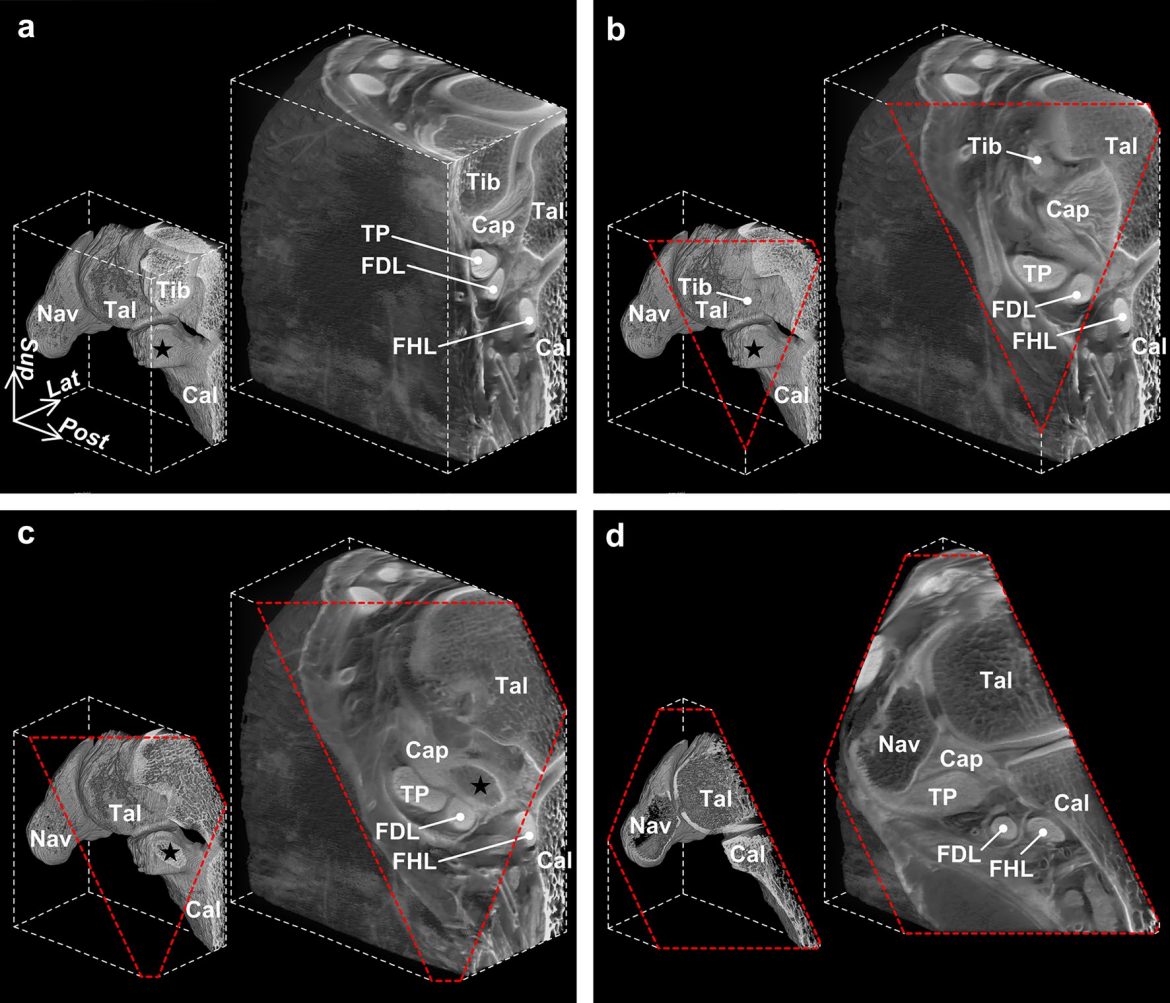
Three-dimensional reconstruction images of the ankle joint capsule complex with flexor tendons. Posteromedial aspect of the right foot. Each view direction of the image on the lower left side corresponds to those on the right side. Red dotted lines indicate the image cutting planes. Medial part (**a**, **b**), extending distally from the tibia (Tib), and plantar part (**c**, **d**), between the sustentaculum tali (star) and navicular (Nav), of the ankle joint capsule (Cap) form a complex with the tendinous sheath of the tibialis posterior (TP) and flexor digitorum longus (FDL). *Cal* calcaneus, *FHL* flexor hallucis longus, *Tal* talus, *Lat* lateral, *Post* posterior, *Sup* superior.

**Figure 2:**
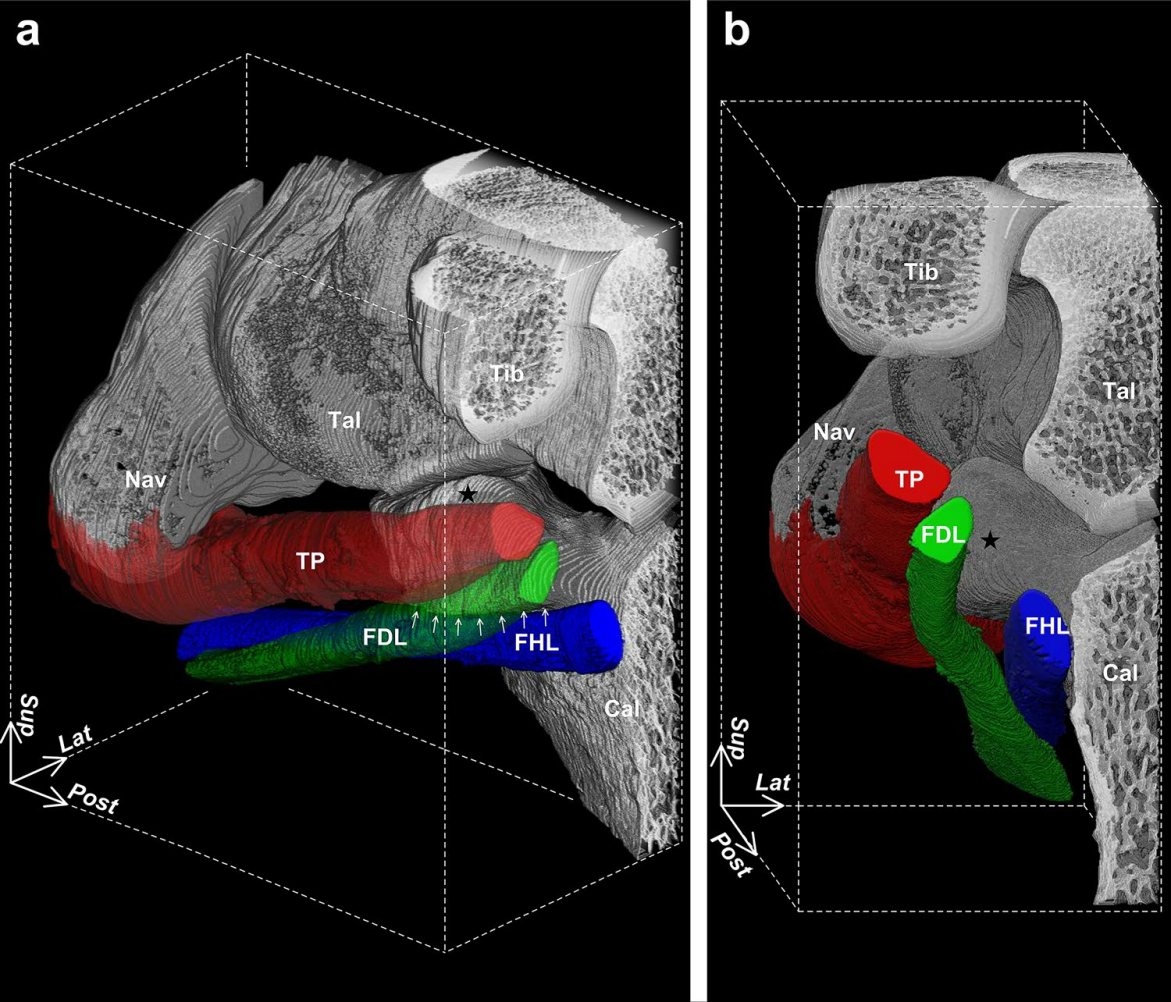
Three-dimensional positional relationships between the flexor tendons and sustentaculum tali. Posteromedial (**a**) and posterior (**b**) aspects of the right foot. The tibialis posterior (TP) and flexor digitorum longus (FDL) in (**a**) are transparent to show the appearance of the sustentaculum tali (star). Arrows in (**a**) indicate the inferior margin of the sustentaculum tali. The FDL runs along the inferomedial part of the sustentaculum tali and is directed to the lateral side of the foot after passing through it. *Cal* calcaneus, *FHL* flexor hallucis longus, *Nav* navicular, *Tal* talus, *Tib* tibia, *Lat* lateral, *Post* posterior, *Sup* superior.

Based on the 3D micro-computed tomography (CT) image, the tendinous sheath of the FDL, along with that of the TP, formed a complex with the medial to plantar part of the ankle joint capsule, which covered the TCN-j (Fig. 1). When primarily focusing on the relations between the FDL and sustentaculum tali, FDL ran anteroinferiorly along the inferomedial part of the sustentaculum tali (Fig. 2a). At the inferomedial aspect of the sustentaculum tali, the course of the FDL was made slightly convex medialward by the bony prominence of the sustentaculum tali and directed to the lateral side of the foot after passing through it (Fig. 2b).

### Histological analysis of relationships between FDL and sustentaculum tali

**Figure 3:**
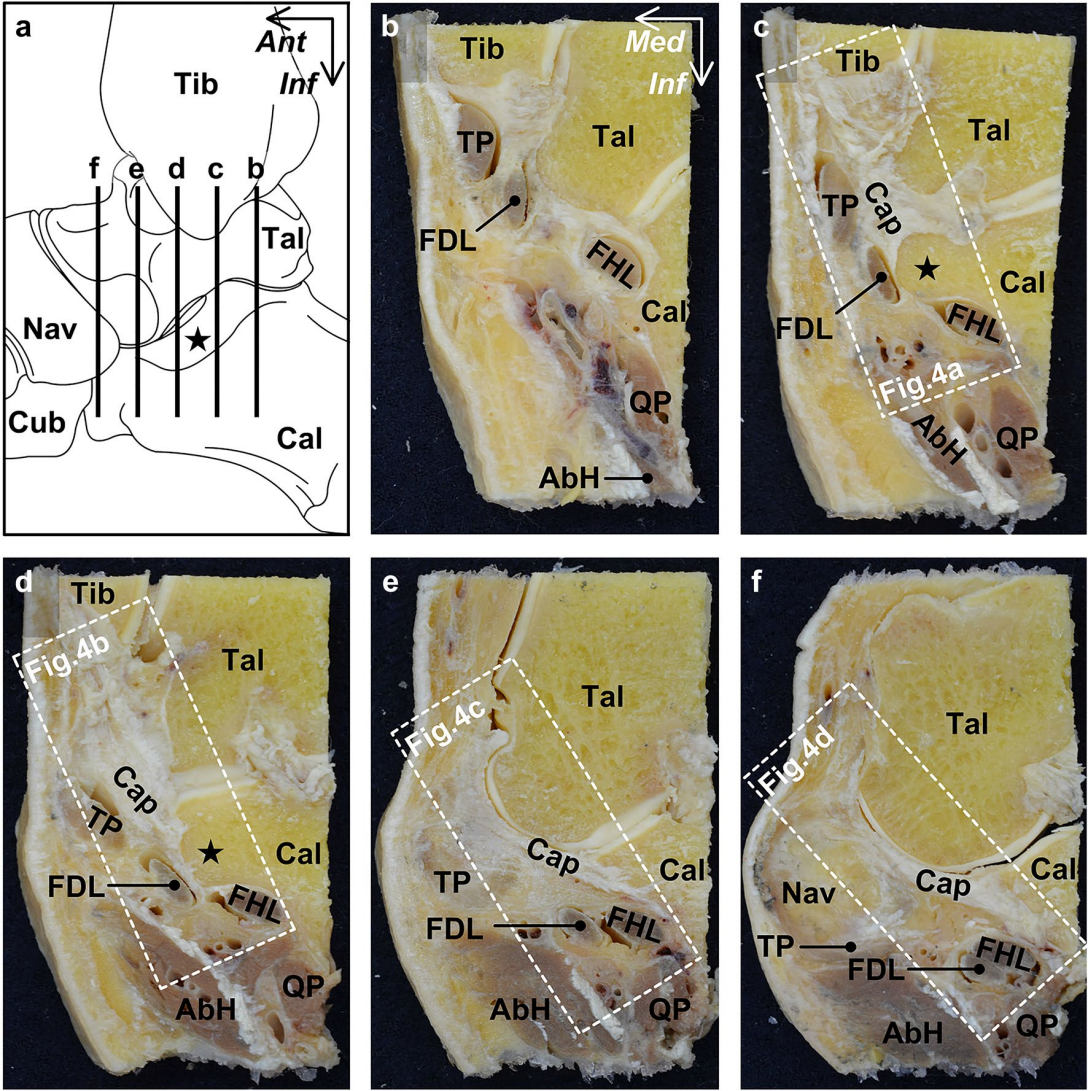
Serial coronal sections of the ankle joint capsule complex with flexor tendons. Schematic illustration (**a**), the medial aspect of the right ankle, indicates the locations of each section, which are shown from (**b**) to (**f**). The boxed regions indicate the selected parts for histological analysis, as shown in Fig. 4. *AbH* abductor hallucis, *Cal* calcaneus, *Cap* joint capsule, *Cub* cuboid, *FDL* flexor digitorum longus, *FHL* flexor hallucis longus, *Nav* navicular, *Tal* talus, *Tib* tibia, *TP* tibialis posterior, *QP* quadratus plantae, *Star* sustentaculum tali, *Ant* anterior, *Inf* inferior, *Med* medial.

**Figure 4:**
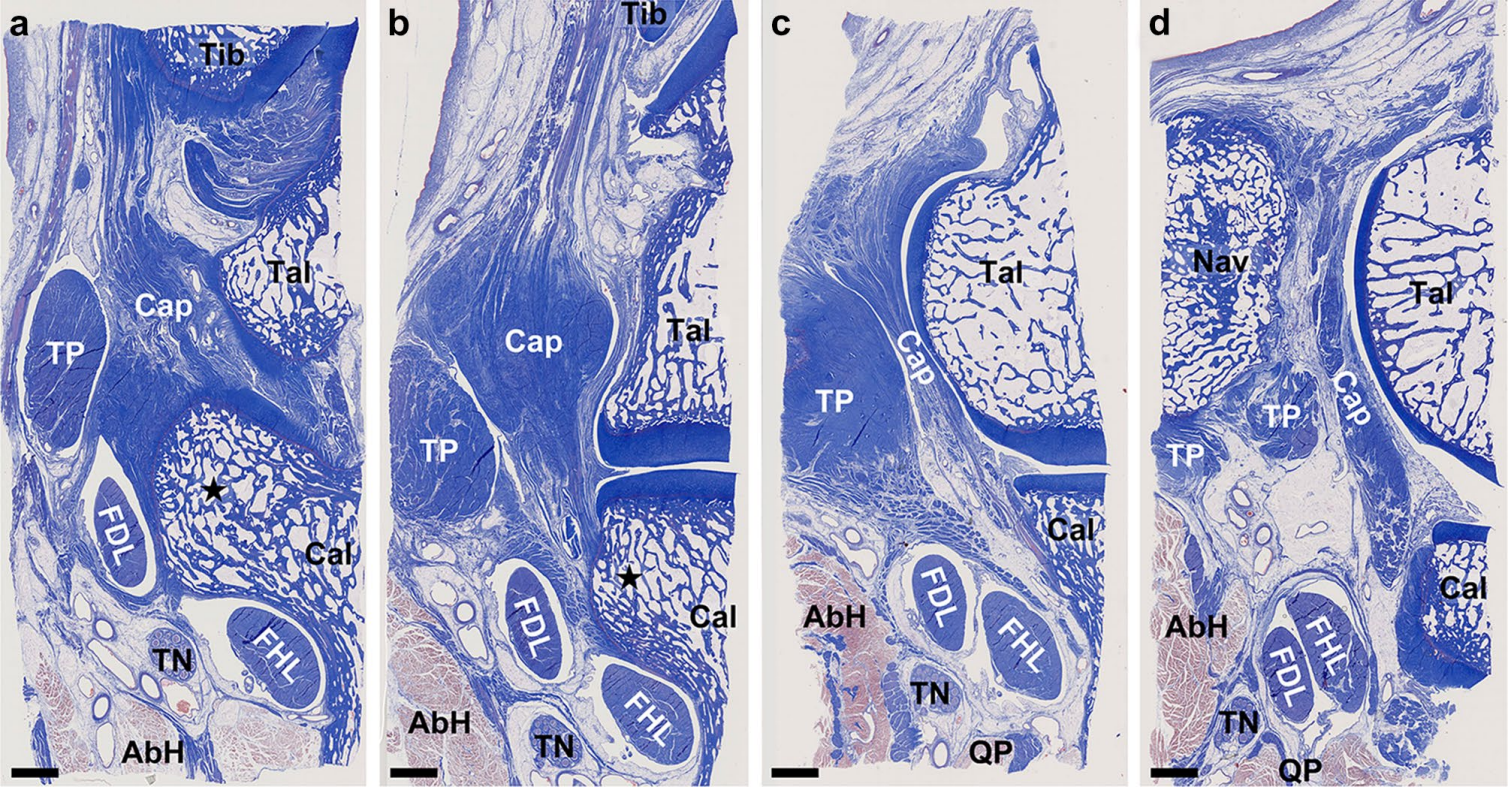
Histological analysis of the ankle joint capsule complex with flexor tendons (Masson’s trichrome staining). Histological sections of the boxed region in Fig. 3c–f are shown in (**a**–**d**). The flexor digitorum longus (FDL) locates inferomedial to the sustentaculum tali (star) (**a**, **b**). Its tendinous sheath, composed of dense connective tissue, attaches from posterior (**a**) to anterior (**b**) part of the sustentaculum tali, in complex with the joint capsule (Cap). Its sheath is partly connected to the tibialis posterior (TP) at the anterior edge of the sustentaculum tali (**c**) and is separated from the TP by loose connective tissue anterior to the sustentaculum tali (**d**). *AbH* abductor hallucis, *Cal* calcaneus, *FHL* flexor hallucis longus, *Nav* navicular, *Tal* talus, *Tib* tibia, *TN* tibial nerve, *QP* quadratus plantae.

To understand the histologic configuration of the connective tissues surrounding the FDL and sustentaculum tali, we analyzed four regions of the coronal ankle sections (Fig. 3). At the posterior (Fig. 4a) and anterior (Fig. 4b) part of the sustentaculum tali, the FDL was found to be inferomedial to the sustentaculum tali, surrounded by a tendinous sheath of dense connective tissue. Additionally, the tendinous sheath of the FDL, along with that of the TP, was attached to the sustentaculum tali, forming a complex with the joint capsule. At the anterior edge of the sustentaculum tali, the FDL and flexor hallucis longus (FHL) were surrounded by the common tendinous sheath (Fig. 4c), which was slightly apart from the anterior edge of the sustentaculum tali. At this level, the border between the TP and its tendinous sheath was unclear and, it appeared that the TP was partly connected to the common tendinous sheath of the FDL and FHL via the tendinous sheath of the TP. At the posterior edge of the navicular and anterior to the sustentaculum tali, the common tendinous sheath of the FDL and FHL was separated from the TP by loose connective tissue (Fig. 4d).

### In vivo ultrasound image analysis of relationships between FDL and sustentaculum tali

**Figure 5:**
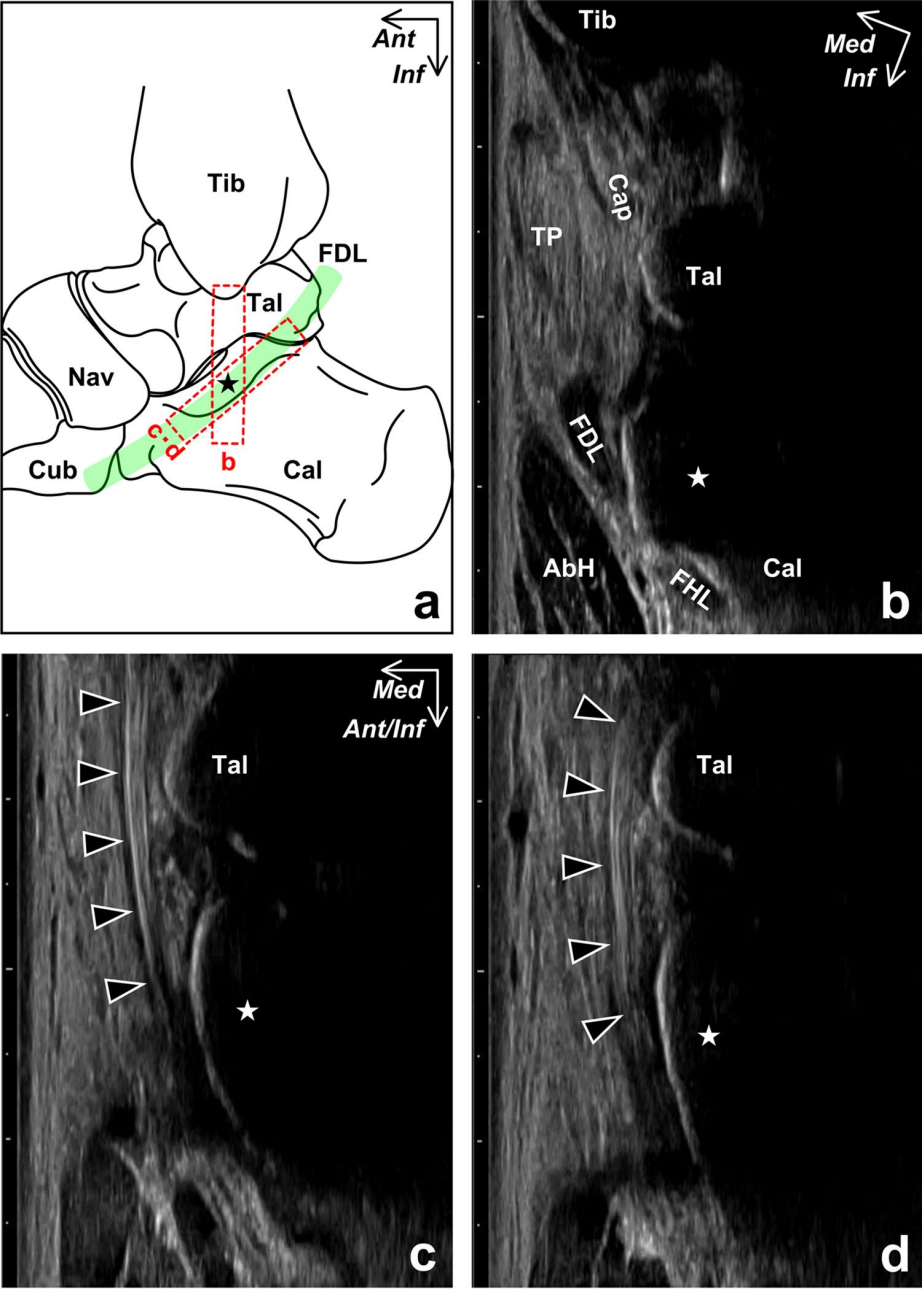
In vivo ultrasound images of the flexor digitorum longus in sitting (without the foot being placed on the ground) and standing. Schematic illustration (**a**), the medial aspect of the right ankle, indicates the ultrasound probe location (red dotted boxes) for visualizing the short (**b**) and long (**c**, **d**) axis images of the flexor digitorum longus (FDL). The FDL locates inferomedial to the sustentaculum tali (star) (**b**), and its curvature of the running course (arrowheads) slightly increased from sitting (**c**) to standing (**d**). *AbH* abductor hallucis, *Cal* calcaneus, *Cap* joint capsule, *Cub* cuboid, *FHL* flexor hallucis longus, *Nav* navicular, *Tal* talus, *Tib* tibia, *TP* tibialis posterior, *Ant* anterior, *Ant/Inf* anteroinferior, *Inf* inferior, *Med* medial.

**Figure 6:**
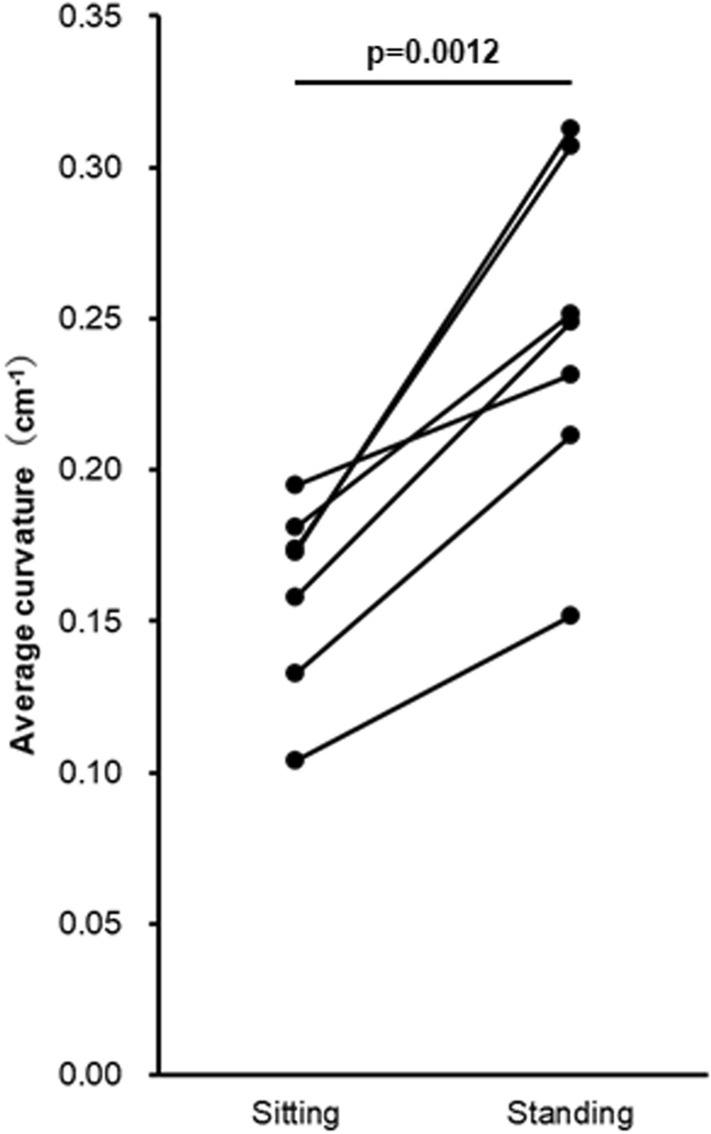
Comparison of the average curvature of the flexor digitorum longus in sitting (without foot being placed on the ground) and standing. The curvature in standing was significantly larger than that in sitting (paired t-test, p = 0.0012).

In vivo ultrasound image confirmed that the FDL was located inferomedial to the sustentaculum tali based on its shot axis image in the coronal plane, and the positional relationships between the FDL and sustentaculum tali were consistent with the anatomic specimen analysis based on the 3D micro-CT image and histological sections (Fig. 5a,b). From sitting (without foot on the ground) to standing, although its positional relationships were maintained, the curvature of the FDL course slightly increased as a result of a calcaneal eversion relative to the talus (Fig. 5c,d). Its average curvature was significantly larger in the standing position (0.24 ± 0.06 cm^−1^) than in the sitting position (0.16 ± 0.03 cm^−1^) (p = 0.0012, Fig. 6).

## Discussion

3D image analysis revealed the FDL to be located inferomedial to the sustentaculum tali. Histologically, the FDL tendinous sheath was attached to the sustentaculum tali in a complex with the TCN-j capsule and was partly connected to the TP at the anterior edge of the sustentaculum tali where the sheath was slightly apart from the sustentaculum tali. Based on the in vivo ultrasound image, the curvature of the FDL course in the standing position was significantly larger than that in the sitting position; however, its positional relation to the sustentaculum tali was maintained.

Previous anatomical descriptions about the positional relationships between the FDL and sustentaculum tali were not clearly described in past articles and textbooks^11–15^. This lack of clarity may be related to the rarity of studies demonstrating its 3D positional relationships and the almost ignored the relationship to the FDL tendinous sheath, which contributed to the maintenance of the FDL location. In the present study, the 3D micro-CT image revealed that the FDL with its tendinous sheath was located inferomedial to the sustentaculum tali. In addition, because the bony groove inferior to the sustentaculum tali, through which the FHL passes, is well-known as the “groove for the tendon of the FHL”^21^, this anatomical knowledge may obscure the importance of the positional relationship between the FDL and sustentaculum tali in these previous reports^11–15^.

With regard to the histological study on the surrounding structure of the TCN-j, Amaha et al. have already revealed that the TCN-j capsule formed the complex with the tendinous sheath of the TP and FDL^9^. However, because their histological sections were parallel to the TP tendon, the comprehensive relationships between the ankle joint capsule and flexor tendons, as shown in this study, have not been histologically shown. The present study revealed that the FDL tendinous sheath was attached to the sustentaculum tali in a complex with the joint capsule and TP was partly connected to the sheath immediately distal to the sustentaculum tali. In other words, we interpreted that the FDL location relative to the sustentaculum tali was fixed by its tendinous sheath, which was attached to the sustentaculum tali or connected by the TP.

**Figure 7:**
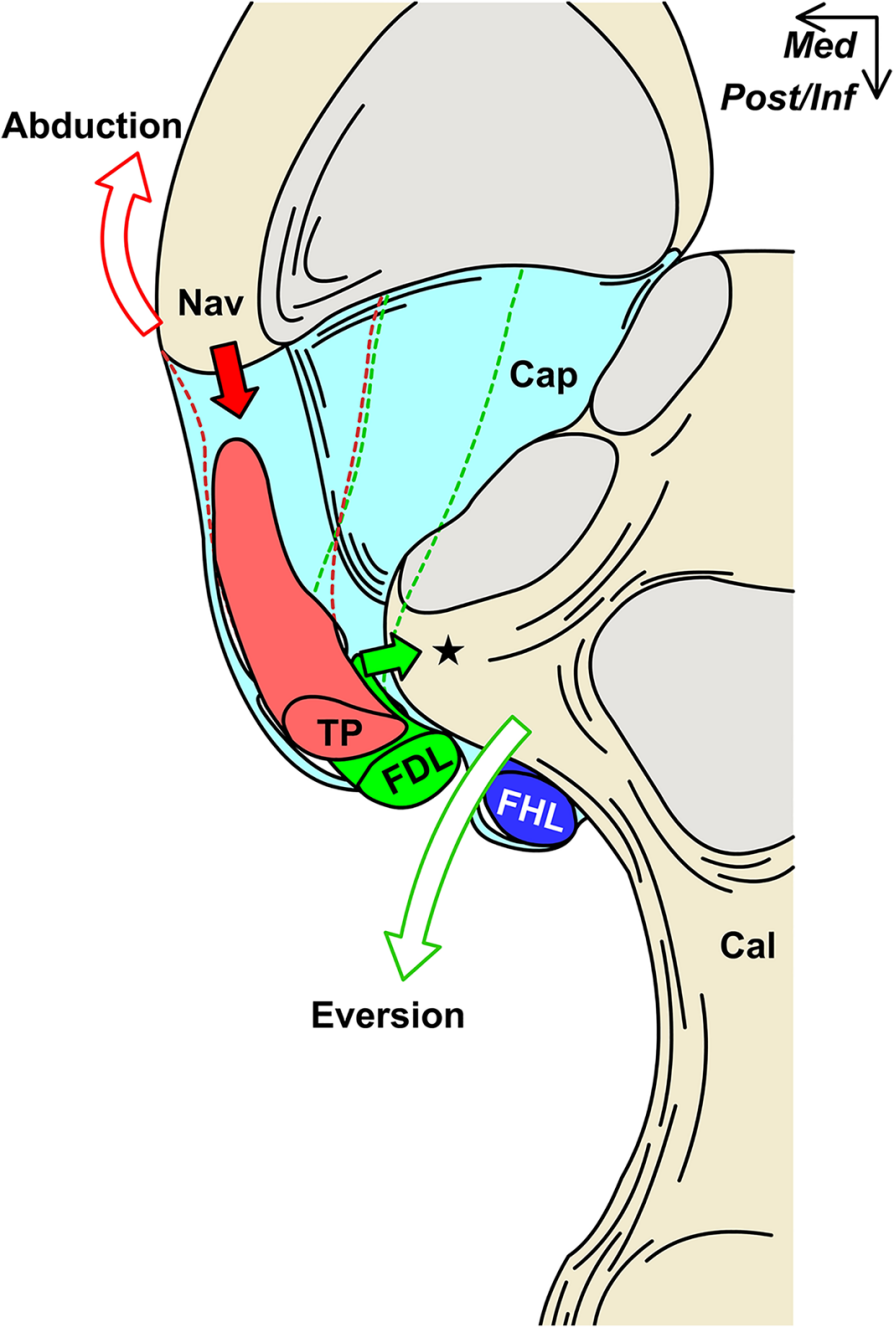
Possible role of the flexor digitorum longus via the joint capsule for talocalcaneonavicular joint (TCN-j) stability. Schematic illustration of the posterosuperior aspect of the TCN-j without the talus. Not only can the pulling force (red filled arrow) of the tibialis posterior (TP) acting on the navicular (Nav) coordinate the navicular abduction (red open arrow), but the flexor digitorum longus (FDL) force against the bending moment (green filled arrow) may also coordinate the calcaneus eversion (green open arrow). These acting force on the TCN-j may be coordinated by the joint capsule (Cap). *Cal* calcaneus, *FHL* flexor hallucis longus, *Star* sustentaculum tali, *Med* medial, *Post/Inf* posteroinferior.

Although recent in vivo studies have been clarified the weight-bearing effect on the bony alignment of the TCN-j^22–25^, the in vivo study about the weight-bearing effect on its soft tissue was limited^26,27^. Moreover, its effect on the FDL has been rarely focused on because of the importance given conventionally to TP or ankle ligaments such as the spring ligament. Our in vivo ultrasound image analysis revealed that the FDL’s course in the standing position was significantly more curved than that in the sitting position. Because the FDL’s location relative to the sustentaculum tali was fixed by its tendinous sheath, as shown in the anatomic specimen analysis, the eversion of calcaneus relative to the talus during weight-bearing might change its curvature via the medial-ward collapsing force of the sustentaculum tali (Fig. 7).

Our study findings are clinically relevant. Because the sustentaculum tali is everted relative to the talus and received the concentrated stress during weight-bearing^19,20,24^, its supporting structure from the inferomedial aspect may be essential to support TCN-j stability in weight-bearing. Based on our findings, the FDL was located inferomedial to the sustentaculum tali, and its curvature was significantly affected by weight-bearing. Therefore, the FDL can affect the inferomedial movement of the sustentaculum tali, and the force against the bending moment may coordinate the eversion of the calcaneus, which is one of the elements in 3D foot deformity of the progressive collapsing foot deformity or adult acquired flatfoot deformity^1,2,6^ (Fig. 7). Furthermore, as the FDL tendinous sheath is attached to the sustentaculum tali in a complex with the TCN-j capsule along with the TP tendinous sheath, it may aid the conventional spring ligament’s contribution to TCN-j stability for maintaining the longitudinal arch^10^ and supporting the midfoot^2,27^. Based on this theoretical interaction, the conventional spring ligament may dynamically coordinate the TCN-j alignment by transmitting the action of the FDL and TP via their tendinous sheaths.

Our study had several limitations. First, if this study had also included pathological conditions, we could have examined the alterations in the FDL anatomy in them, as compared to the regular joints. Therefore, it is unclear whether the role of the FDL presented in this study could also be observed in pathological conditions, and this warrants verification in clinical situations. Although the clinical relevance that we presented may not be immediately applied in the clinical setting, such as surgery or diagnosis, it should be essential when considering TCN-j stability. Second, the sample size was relatively small. However, the effect size and power of the ultrasonographic data were 0.92 and 0.89, which were good enough. Moreover, we compensated for the small sample size with a multidimensional analysis, including macroscopic, histological, and ultrasound analysis. Finally, the advanced age of the individuals from whom the anatomic specimens were obtained might have affected our findings. Nevertheless, the effect was expected to be minimal because the anatomic specimen findings were almost verified by ultrasonography.

In conclusion, the FDL was located inferomedial to the sustentaculum tali. Additionally, its tendinous sheath was attached to the sustentaculum tali in complex with the TCN-j capsule, and its curvature was significantly larger in the standing position than in the sitting position. This anatomical knowledge may provide a better understanding of TCN-j stability.

## Methods

### Anatomic specimen preparation

For this study, six ankles from six Japanese body donors (three males and three females; mean age at death: 64.5 years) that were donated to the Department of Anatomy at Tokyo Medical and Dental University were used. Prior to their death, all donors voluntarily declared that their remains would be donated for education and study. Our study complied with the Japanese law entitled “Act on Body Donation for Medical and Dental Education,” and the study design was approved by the Medical Research Ethics Committee of Tokyo Medical and Dental University (approval no.: #M2020-382). Additionally, all procedures were performed in accordance with the Japanese guidelines entitled “Ethical Guidelines for Medical and Health Research Involving Human Subjects”. Anatomic specimens that had no apparent osteoarthritic changes and did not undergo foot and ankle surgery during their lifetime were included in the study.

All anatomic specimens were fixed with 8% formalin and preserved in 30% ethanol. Using a diamond saw (EXAKT 312; EXAKT Advanced Technologies, Norderstedt, Germany), the ankle specimens were obtained by cutting immediately superior to the inferior edge of the medial malleolus parallel to the foot plantar surface. In addition, parts of the specimens, which were anterior and lateral to the navicular, and inferior one-third of the calcaneus, were cut and removed using the diamond saw. To determine if any bony or soft tissue abnormalities were present, the sectioned specimens underwent micro-CT (inspeXio SMX-100CT; Shimadzu, Kyoto, Japan) with a 200-µm resolution. From the series of micro-CT images, Computer-aided design (CAD)-3D models were also obtained using ImageJ software version 1.53 (National Institutes of Health, Bethesda, MD, USA). Based on the micro-CT image series and also a secondary check of CAD-3D models, no apparent bony abnormalities were observed in all six ankles. Therefore, three ankles were randomly assigned to the 3D image analysis using phosphotungstic acid enhanced micro-CT, and the remaining three to histological analysis.

### 3D image analysis of the ankle using phosphotungstic acid enhanced micro-CT

Three ankles (one male and two females; mean age at death: 64.7 years) were analyzed by the phosphotungstic acid enhanced micro-CT using the previously described method, as follows^28–31^. Phosphotungstic acid enhanced micro-CT analysis could acquire the 3D information of bone and soft tissue^28–31^. The specimens were dehydrated in 70% ethanol solutions for one day and then stained with 1% phosphotungstic acid solution in 70% ethanol for 12–15 weeks. The stained specimens were subsequently scanned using micro-CT at an image pixel size of 50 μm with 1024 × 1024 pixels. The scanned images were reconstructed using the “Volume Viewer” plugin of ImageJ software, in which the “Distance Slider” function could cut and observe the voxel image at any cross-section. Additionally, using ImageJ, binarized sequential images of the bony elements and flexor tendons were individually created and then combined into one sequential image to visualize the 3D relationships between the flexor tendons and sustentaculum tali.

### Histological analysis of the coronal sections of the ankle

Three specimens (two males and one female; mean age at death: 64.3 years) assigned for histological analysis were embedded in a 3% agar solution and frozen at − 80 °C. The specimens were then serially sectioned into 5-mm thick segments in the coronal plane using a band saw (WN-25-3; Nakajima Seisakusho, Osaka, Japan). Each axial section level was identified based on the correspondence between the axial section bony morphology and that observed on the micro-CT image at the anatomic specimen preparation. After removing the excess agar, we chose coronal sections at the following four levels: at the posterior and anterior part of the sustentaculum tali, its anterior edge, and the posterior edge of the navicular. Subsequently, we harvested a block from each coronal section, including the flexor tendons, joint capsule, and sustentaculum tali or navicular; the blocks were decalcified for three weeks with Plank–Rychlo solution (AlCl_3_:6H_2_O [70.0 g/L], HCl [85.0 mL/L], and HCOOH [50.0 mL/L])^32^. After decalcification, each block was dehydrated and embedded in paraffin. The paraffin-embedded tissue was sliced into 5-μm sections with a 1-mm interval; the sections were stained using the Masson trichrome staining protocol.

### In vivo ultrasound image analysis of the ankle

Ten ankles of ten healthy volunteers (five males and two females; mean age, 21.6 years; height, 1.7 ± 0.1 m; weight, 67.3 ± 12.4 kg) were investigated. We recruited individuals with no history of previous foot or ankle surgery and without apparent medial longitudinal arch collapse, hindfoot valgus, or forefoot abduction. The study design was also approved by the ethics committee of Morinomiya University of Medical Sciences (approval no.: #2021-081). All procedures were performed in accordance with the Declaration of Helsinki (last modified in 2013) and the Japanese guidelines entitled, “Ethical Guidelines for Medical and Health Research Involving Human Subjects”. All potential participants were informed of the study requirements, benefits, and risks, after which those desiring to participate in the study provided written informed consent.

Ultrasound assessment of the ankle was performed using an ultrasound scanner (SNiBLE; Konica Minolta, Tokyo, Japan) with a 4–18 MHz linear transducer. A short-axis B-mode image of the FDL was visualized at the dorsiflexion 0 degrees in the coronal plane, with the medial malleolus, talus, and sustentaculum tali. Subsequently, by rotating the transducer slightly, the long axis image of the FDL along its running course was visualized in the sitting (without foot on the ground) and standing positions. Based on recorded long axis images of the FDL, the average curvature of the running course was quantitively analyzed by using the “Kappa” function of the Image J software to assess the morphological difference between sitting and standing. A region of interest was set along the medial margin of the FDL from the point where the talus protruded most medially to most distal FDL part that can be visualized. To assess the reliability, visualization of the long axis FDL image and measurement were performed by two examiners, and the average of two measurements were recorded for statistical analysis. Intraclass correlation coefficients were calculated to determine the inter-rater reliability of each measured value.

### Statistical analyses

Statistical analyses were performed using SPSS software version 27.0 (IBM Corp., Armonk, NY, USA). Distributions of all measurements consistently passed the Shapiro–Wilk test, and the data were expressed as mean and standard deviation. Statistical comparisons of the FDL average curvature in sitting and standing were performed using the paired t-test, and the significance level was set at p < 0.05. In addition, using G*Power software version 3.1.9.6, the effect size was calculated, and post-hoc power analysis was performed. The effect size and power were 0.92 and 0.89, respectively.

Intraclass correlation coefficients were also determined using a measurement process analysis. The qualitative cut-offs for intraclass correlation coefficients values are reported as follows: poor, < 0.40; fair, 0.40–0.59; good, 0.60–0.74; and excellent, 0.75–1.0^33^. Intraclass correlation coefficients of the average curvature in sitting and standing were 0.96 [95% CI 0.75–0.993] and 0.98 [95% CI 0.90–0.997], respectively. All intraclass correlation coefficients were ≥ 0.75 (range, 0.75–0.997), indicating excellent agreement.

## Data Availability

The datasets used and/or analyzed during the current study are available from the corresponding author upon reasonable request.
